# Clinical Coders' Perspectives on Pressure Injury Coding in Acute Care Services in Victoria, Australia

**DOI:** 10.3389/fpubh.2022.893482

**Published:** 2022-06-01

**Authors:** Carolina Dragica Weller, Louise Turnour, Elizabeth Connelly, Jane Banaszak-Holl, Victoria Team

**Affiliations:** ^1^Faculty of Medicine, Nursing and Health Sciences, School of Nursing and Midwifery, Monash University, Clayton, VIC, Australia; ^2^Cabrini Hospital, Malvern, VIC, Australia; ^3^Faculty of Medicine, Nursing and Health Sciences, School of Public Health and Preventive Medicine, Monash University, Melbourne, VIC, Australia; ^4^Monash Partners Academic Health Science Centre, Clayton, VIC, Australia

**Keywords:** clinical coders, quality assurance–healthcare, clinical records documentation, pressure injury (ulcer), pressure injury documenting, acute care services, electronic medical record (EMR), coding standard

## Abstract

Pressure injuries (PIs) substantively impact quality of care during hospital stays, although only when they are severe or acquired as a result of the hospital stay are they reported as quality indicators. Globally, researchers have repeatedly highlighted the need to invest more in quality improvement, risk assessment, prevention, early detection, and care for PI to avoid the higher costs associated with treatment of PI. Coders' perspectives on quality assurance of the clinical coded PI data have never been investigated. This study aimed to explore challenges that hospital coders face in accurately coding and reporting PI data and subsequently, explore reasons why data sources may vary in their reporting of PI data. This article is based upon data collected as part of a multi-phase collaborative project to build capacity for optimizing PI prevention across Monash Partners health services. We have conducted 16 semi-structured phone interviews with clinical coders recruited from four participating health services located in Melbourne, Australia. One of the main findings was that hospital coders often lacked vital information in clinicians' records needed to code PI and report quality indicators accurately and highlighted the need for quality improvement processes for PI clinical documentation. Nursing documentation improvement is a vital component of the complex capacity building programs on PI prevention in acute care services and is relied on by coders. Coders reported the benefit of inter-professional collaborative workshops, where nurses and coders shared their perspectives. Collaborative workshops had the potential to improve coders' knowledge of PI classification and clinicians' understanding of what information should be included when documenting PI in the medical notes. Our findings identified three methods of quality assurance were important to coders to ensure accuracy of PI reporting: (1) training prior to initiation of coding activity and (2) continued education, and (3) audit and feedback communication about how to handle specific complex cases and complex documentation. From a behavioral perspective, most of the coders reported confidence in their own abilities and were open to changes in coding standards. Transitioning from paper-based to electronic records highlighted the need to improve training of both clinicians and coders.

## Introduction

Pressure injuries (PIs) substantively impact quality of care during hospital stays, although only when they are severe or acquired as a result of the hospital stay are they reported as quality indicators. The three main Australian sources of PI data include: (1) incident reporting systems, (2) clinical coded data derived from medical records and discharge summaries, and (c) data generated from Pressure Ulcer/Injury Point Prevalence Surveys (PUPPS/PIPPS) (1). Australian researchers (1–4) have repeatedly highlighted the lack of consistency and uniformity in the reporting of hospital-acquired pressure injury (HAPI), which leads to inaccurate data interpretation. Furthermore, these researchers have suggested that there is a need for greater uniformity of reporting and data standardization before providers can benchmark performance across hospitals and evaluate time trends in PI incidence. This study examines challenges that hospital coders face in accurately coding and reporting PI data and subsequently, explores reasons why data sources may vary in their reporting of PI data.

A PI is defined as “localized damage to the skin and/or underlying tissue, as a result of pressure or pressure in combination with shear” (5). The National Pressure Ulcer Advisory Panel (NPUAP), the European Pressure Advisory Panel (EPUAP) and Pan Pacific Pressure Injury Alliance (PPPIA) (5) identify four stages of increasing severity in PIs: stage I—non-blanchable erythema, stage II—partial thickness skin loss, stage III—full thickness skin loss, and stage IV—partial thickness tissue loss. In addition, a PI is classified as either an unstageable PI, when eschar or slough obscure the assessor's ability to determine the true depth of the injury, or suspected deep tissue injury (SDTI), when a localized area is of discolored purple or maroon colors (5). The depth of tissue damage may vary, which is related to anatomical location (5).

PIs may be present and detected at hospital admission, or they can occur at any point during the patients' admission in acute care. A PI acquired during a hospital stay is referred to as a hospital-acquired pressure injury (HAPI). Multiple factors on various levels may increase the risk of HAPIs occurring. For individual patients, key contributing factors are whether the patient is advanced in age, or has multiple comorbidities or high functional and mobility dependency upon admission. Physiologically, necessary factors include whether there is high skin perfusion and low oxygen saturation levels. Hospital-episode specific factors, such as whether the hospital stay is prolonged and the presence of suboptimal nurse-to-patient ratios, also have been reported to increase PI incidence (6–8).

Globally, intensive care unit (ICU)-acquired prevalence of PI was reported at 16.2% (95% CI 15.6–16.8); the study included 1117 ICUs in 90 countries (9). However, countries vary in the reported ICU-acquired prevalence of PIs, which is attributed to the organizational and workforce factors, including HAPI prevention protocols, the use of preventive measures, staffing levels, and the quality of care (8). A 2019 Australian study conducted at eight tertiary hospitals included 1,047 patients aged ≥65 years with limited mobility, the authors reported 10.8% of participants developed a PI within the first 36 h of hospital admission (6).

The incidence and prevalence of PI are projected to increase in upcoming years due to global population aging, increasing incidence of chronic illness, and increasing dependency levels, and particularly of concern is the potential for increases in HAPI, which hospitals have long sought to reduce. For example, incidence of HAPI during COVID-19 has been linked to the prone positioning needed for COVID-19 patients with acute respiratory distress syndrome (7, 10–15). HAPI are associated with poor health outcomes (16), reduced quality of life, and significant healthcare costs (17), particularly for those with stages III and IV PIs, which may represent approximately one third of the total costs for HAPI (18). Accurate PI staging and early prevention are important to monitoring and benchmarking hospital quality of care and associated care costs.

Globally, researchers (16, 19) have repeatedly highlighted the need to invest more in quality improvement, risk assessment, prevention, early detection, and care for PI to avoid the higher costs associated with treatment of deep tissue injuries. Quality improvement requires complex but sustainable approaches (20–22) based in a capacity building framework (19, 23) that includes use of high quality data. Quality of PI data can be impacted by various challenges related to PI identification, classification, measurement, and reporting (2, 24), including the accuracy of clinical documentation (25) and factors related to work of coders.

Australian inpatient hospital admissions receive a single Diagnosis-Related Group (DRG) code that is subsequently used by payers to process healthcare providers' claims and is used for hospital-based outcome indicators. The appropriate DRG for an inpatient admission is determined from patient records manually and standardized to ICD format (26). DRGs are then assigned using the current edition of the International Classification of Disease ICD-10-AM/ACHI/ACS Eleventh Edition (https://www.ihpa.gov.au/what-we-do/icd-10-am-achi-acs-current-edition). The Australian Commission on Safety and Quality in Health Care promotes improved documentation leading to DRGs in the National Safety and Quality Health Service Standards, which are the basis for the country's hospital-based outcome indicators (25). Within the Australian healthcare system, inpatient episodes assigned to PI treatment can receive optimal funding from payers.

To understand clinical coders' behavior related to PI coding, we used the Theoretical Domains Framework or TDF (27) to frame a study into the challenges that hospital coders face as they seek to accurately and consistently reporting PIs and PI staging. The TDF is widely utilized by researchers as a theory-informed approach to analyzing behavioral determinants when process implementation is problematic. This study applies Atkins et al. (28) version (28) of the TDF with 14 domains of challenges: (1) Knowledge of the process, (2) Individual skills with the process, (3) Beliefs about one's own capabilities, (4) Beliefs about consequences, (5) Environmental context and resources, (6) Social influences, (7) Behavioral regulation, (8) Optimism, (9) Emotions, (10) Goals, (11) Social/professional role and identity, (12) Reinforcement, (13) Intentions, and (14) Memory, attention and decision-making capability. Originally, Michie et al. (27) developed the TDF to explain who follows evidence-based guidelines, but Atkins et al. (28) have generalized the framework for use across implementation issues.

Coders' perspectives on quality assurance of the clinical coded PI data have never been investigated. However, a number of previous studies suggest that the TDF will be a useful framework for understanding the complexity of coding PIs and what factors impact how coders engage in PI coding. For example, recent Canadian studies (29, 30) of coders' perspectives on quality assurance reported the following barriers to producing high-quality medical coding data: (1) clinicians' notes can be incomplete and nonspecific; (2) errors and discrepancies can be present in patients' charts; (3) discrepancies in clinicians' and coders' terminology are present; (4) coders have a limited role in questioning, interpreting and modifying a diagnosis; (5) coder-clinician communication issues are present; and 6) staffing issues can occur. The identified barriers are well linked with the TDF domains and the related constructs. Quality assurance research examines the process used to meet optimal standards (31); and the past studies mentioned have identified a number of barriers that could make it difficult for medical coders to provide optimal coding of PI cases. Conducting this study, we aimed to identify individual, organizational and health system level barriers to the optimal PI coding process.

## Materials and Methods

### Aim/s and Objectives

This article is based upon data collected as part of a multi-phase collaborative project to build capacity for optimizing PI prevention across Monash Partners health services (23). One of the project objectives was to identify individual, organizational and health system level barriers to the optimal PI coding process. Other objectives are presented in Table 1. The detailed description of Monash Partners Capacity Building Framework has been discussed elsewhere (23).

**Table 1:** Monash partners capacity building project: project objectives.

**Project Phase**	**Project objectives**
Phase 1	1. Map and compare existing PI data across four MP health services (Alfred Health, Cabrini, Monash and Peninsula Health). 2. Develop and pilot PI data harmonization approach across Alfred Health, Cabrini, and Peninsula Health. 3. Identify alignment of PI assessment tool/s and PI coding definitions. 4. Standardize risk adjustment procedures to account for differences in risk of PI development. 5. Establish and evaluate the cost effectiveness of pilot PI clinical registry.
Phase 2	1. Identify individual, organizational and health system level barriers to integrate PI assessment and care across the continuum. 2. Interview and develop training modules for nurses and clinical coders, based on interviews to ensure accurate PI assessment, documentation and coding across Monash Partners hospitals.

### Methods

This qualitative study uses data from 16 semi-structured phone interviews with clinical coders and was part of a larger study including 48 total semi-structured phone interviews also including nurses from four acute care hospitals in Melbourne Australia. All interviews were audio-recorded using a handheld mobile recording device. Participant verbal consent to both the interview and audio recording was obtained prior to each interview.

### Recruitment Strategy

Participants were recruited with the support of the project Advisory Committee, which had representatives from the University, four major acute care hospitals participating in this project, Wounds Australia – the National peak body for wound prevention and management, and Monash Partners – a partnership between leading health services, teaching and research organizations, and consumer support group. Representatives of the Advisory Committee from the participating health services verbally explained and provided a brief summary of our project to the clinical coders in each of their health services. Clinical coders wishing to participate then contacted the interviewer (LT) to schedule their interview.

### Data Collection

Data were collected, using an interview guide developed by VT based on the TDF, and refined by LT, CW, JBH, and approved by the Project Advisory Committee. The interview guide (Supplementary File 1) included open-ended questions related to PI coding experience and was guided by the Theoretical Domains Framework (TDF) domains (2017 version). Open-ended questions were followed by prompts that probed the barriers and enablers to optimal PI coding and identified coders' needs and suggestions for improving the process of PI coding. Interview questions were modified by the interviewer (LT) depending on the interview flow. The interview guide was not piloted, because the TDF framework and questions are well supported in previous research.

Phone interviews were conducted by an experienced wound research nurse (LT) between December 2020 and March 2021. LT was employed by Monash University; and had no work-related relationship with the clinical coders recruited from health services. There was no unequal relationship between the interviewer and the participants. The average interview lasted 45 min, ranging between 29 and 64 min, depending on the participant's availability. All audio files were verbatim transcribed using professional transcription, and the first four transcripts were compared to audio-recordings by LT and VT to ensure the accuracy of the transcribed text. Interview transcripts were not sent back to participants for verification. All participants were reimbursed with a $25 Coles Myer gift card for participation, which was posted to their preferred address upon completion of the interview.

### Ethical Considerations

This study was conducted in line with the ethical guidelines of the 1975 Declaration of Helsinki. The University Human Research Ethics Committee approval was obtained for both the main study and a nested qualitative study. The main project was approved by the Alfred Hospital Ethics Committee (Project No: 66/17). Site-specific approvals were received from the participating health services ethics committees.

### Data Analysis

The data were analyzed using the qualitative data analysis software, NVivo Version 12, later upgraded to Version 20.3. We adopted a theory-driven conceptual analysis (32, 33) as the data analysis method. We utilized the TDF (28) to guide analyses, using a coding framework that included all TDF constructs and the 14 TDF domains. VT initially coded the first three transcripts to develop the coding framework. The coding framework was then reviewed by CW and JBH.

We manually created the first and the second level nodes using a deductive approach that matched to the TDF domains (first level) and the TDF constructs (second level). The third level or child nodes were then created inductively to identify specific barriers and enablers to the optimal PI coding process, as well as, current needs and suggestions for coding improvement. Technically, utterances were linked with the particular barrier/enabler or need related to PI coding process, and mapped across the developed coding framework based on the TDF domains and related constructs. Although we were mindful of potential additional themes, we did not find any that were outside of this framework.

We interviewed 16 participants. Data saturation was reached by the 12th interview, when the remaining four voice files were with the transcription agency. We reached saturation when subsequent analysis did not generate any new barriers/enablers and needs/suggestions related to PI coding process within the TDF constructs. We then informed the remaining two coders who had agreed to be interviewed to thank them for their interest and stopped recruitment.

## Results

### Participant Characteristics

Sixteen participants were recruited, including 11 clinical coders from three public health services and five coders from a private health service in Victoria Australia. All participants were female, which reflects the national profile of clinical coders, where 93% of them are female (34). Three participants were in the 25–34 years age group, six in – 35–44, six in – 45–54, and one in – 55–65. Further details on their education and years in clinical practice are provided in Table 2.

**Table 2:** Participant characteristics (*n* = 16).

**Participating health services**		
Public health service 1	4	25%
Public health service 2	3	19%
Public health service 3	4	25%
Private health service	5	31%
**Gender**		
Male	0	
Female	16	100%
**Age**		
25–34	3	19%
35–44	6	37%
45–54	6	37%
55–65	1	6%
**Education**		
Bachelor of Nursing and HIMAA^*^ clinical coder course	1	6%
Bachelor of Education and HIMAA clinical coder course	1	6%
HIMAA clinical coder course	2	13%
Bachelor Nutrition and Dietetics and Masters of Health Information Management	1	6%
Bachelor of Applied Sciences and HIMAA clinical coder course	1	6%
Bachelor of Health Sciences and Masters of Health Information Management	1	6%
Bachelor of Health Sciences and Bachelor of Health Information Management	2	13%
Bachelor of Health Information Management	7	44%
**Current position title**		
Clinical coder	3	19%
Health Information Manager	7	44%
Health Information Manager/coding educator	2	13%
Health Information Manager/coding auditor	3	19%
Health Information Manager/coding educator and auditor	1	6%
**Current involvement in coding-related activities**		
2 days/week	1	6%
3 days/week	4	25%
4 days/week	4	25%
5 days/week	7	44%
**Duration of clinical practice**		
<10 years	7	44%
11–20 years	5	31%
21–30 years	3	19%
>30 years	1	6%
**Coding experience**		
<5 years	3	19%
6–10 years	4	25%
11–20 years	5	31%
>20 years	4	25%
**Completion of course on pressure injury coding**		
Completed	1	6%
Not completed	15	94%

* *HIMAA, The Health Information Management Association of Australia Ltd. is the peak professional body for health information management professionals in Australia*.

### Summary of the TDF Domains

**Figure 1:**
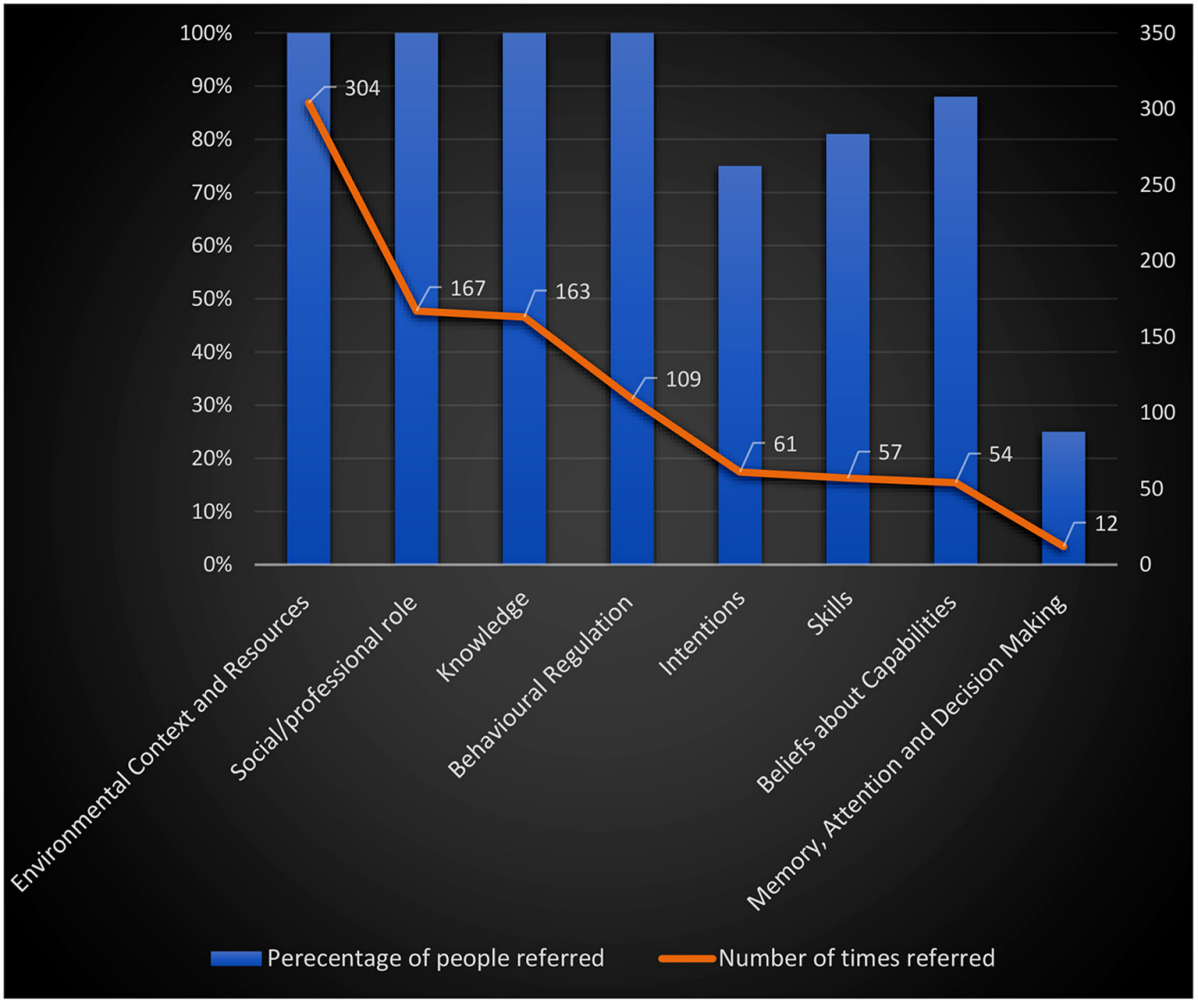
The summary of the TDF domains: percentage of the participants referred and the number of times referred.

Eleven theoretical domains were mentioned in relation to the PI coding process, including barriers, enablers and suggestions for improvement (Table 3). The domains judged to be most important were those referred to most frequently by all participants: Environmental Context and Resources (referred 304 times), Social/professional Role and Identity (referred 167 times), Knowledge (referred 163 times), and Behavioral Regulation (referred 109 times). Other important domains included Intentions (referred 61 times by 12 of participants), Skills (referred 57 times by 13 participants), Beliefs about Capabilities (referred 54 times by 14 participants), and Memory, Attention and Decision Making (referred 12 times by 4 participants) (Figure 1). Less common domains included: Emotions (referred 3 times by 2 participants), Reinforcement (referred 2 times by 2 participants), and Beliefs about Consequences (referred once by 1 participant). Optimism and Goals domains did not emerge. We did not ask specific questions related to coders' goals and their optimism and during interviews, coders did not provide any relevant information related to these domains. Social influences domain was combined with Social/professional Role and Identity domain given that in the hospital setting in which the coders work, their identity and social influences were shaped largely by the clinicians and other professionals with which they interacted. Our decision to combine both domains was based on similarity of what the participants said across these domains.

**Table 3:** Main domains, constructs and sample quotes.

**Domains**	**Main constructs**	**Main sub-themes**	**Sample quotes**
Environmental Context and Resources	Barriers and Facilitators	Accuracy of documentation	Often, you'll get, say, a leg ulcer with peripheral vascular disease with gangrene, with a diabetic foot ulcer, with a pressure injury stage 3… You want to make sure that you've reflected all those conditions in the correct amount of coding, so you're not double-coding and you're coding it correctly…That's what I find quite interesting and that's the challenge. Some cases are so simple and, therefore, like pressure injury stage 3, no problem, code that. It's the ones that are the diabetic feet with pressure injury or is that a blister, is that a wound? And that's what becomes quite challenging… C403 I personally will only code it if it's documented in the notes, and when it is beyond routine care. So, say if it just says a patient has a pressure injury, given air mattress, then I wouldn't code it. I would code it when there's a care plan. C303 Q: Is there any things you'd like changed or – would improve things for your end? A: From a coding point of view, I guess – I've probably said it a million times, just getting the best documentation that we can. Because that will reduce the time we spend trying to dissect all that information and then having to send a query, getting it back, changing the codes, finalizing the codes and then repeating that for the next case. C101
		Electronic medical record (EMR) and coding	Nurses used to write down ‘sacral’, or ‘pressure area on sacrum’, but now, if they haven't put this thing into the skin incision thing, it doesn't even appear in front of us. When something doesn't appear in front of you – other coders don't even know that exists. And there are hundreds and hundreds of things like that all over the EMR. It really has been – I know it's all designed for safety of the patient, but for us it's been truly quite mega. C302 I think the electronic medical record has added complexity because when the nurses documenting it in their view they can't necessarily see what other nurses have documented along the way, or other clinicians have documented along the way. For example, if they're documenting, because these go into the result section now, whereas before it was a clinical note and it would be a note that would be added to and built on by nurses during the stay. Whereas now it's all this discreet data entry where they might not have the context and the awareness of what else is being input. And so, we find that that perpetuates bad data because they never happen across a note, like they would have when it was paper and go, ‘Oh actually our wound care nurses said it's a stage two, not a stage one, I better from now on document it as a stage two’. So, we do find this EMR discreet data sometimes perpetuates bad data entry, particularly in long stays. C102 Now everything has gone to electronic. So, it is more difficult, I suppose, to know where to look and to remember to look in the electronic system, than maybe what it was in the scan system. But it would be there in your face and the form would pop up, whereas the electronic system, unless I go searching for it, well then you don't see it quite so easily. So maybe more are being missed. C402
Social/professional Role and Identity	Organizational Commitment	Auditing	The internal auditing that happens is more for revenue purposes, so it's not so much quality, but you'll improve the quality, I guess, if something gets flagged. So, we have an algorithm actually that runs over the data and prioritizes what is most likely to change and for the biggest revenue change So that's also run over data each day and probably on average five records a day are flagged for auditing. C202 We audit DRGs [Diagnosis Related Groups] and we audit to make sure that they're at the correct DRG and we also have some quality audits. So, we've actually introduced a pressure injury quality audit at my organization. So, anything that is unspecified will get seen by the CNC [Clinical Nurse Consultant] and they do their own research in the record to ascertain whether or not it is a pressure injury. It's like a reconciliation audit. C103
		Support from coding educators	We do have a coding educator who's available 5 days a week who provides significant support and extended training for people in the areas that they're not familiar with. The hospital really does invest in our training quite a lot, and resources. C203 We have a senior coder on duty every day, and you can write to them, put it in the Coding Advisor's box, about anything or anyone. So, it could be: “Do I cancel this? Do you think this can meet 0002?” And you will get your answer back within the day from someone who's highly educated in coding. And that will give you guidance. And then it'll come back with all the reasons, and all the standards that they've looked at. C302 When a coder first starts we do a training program with them of the coding specialty which includes pressure injuries and also access to coding books, the online software. We do run the education program so it's a combination of face-to-face presentations, self-directed learning which we've had a lot of last year with PowerPoint presentations and also quizzes. C201
		Internal meetings	We also have the coding meetings, where we have an option for people to raise coding queries like, “I coded this and I'm not exactly sure. Has anyone come across a situation like this?” C401
		Interprofessional collaboration with clinical staff	Because we've worked with the wound nurses, it becomes a lot easier, but prior to that, there was a lot of confusion and a lot of time taken in terms of writing the documentation queries, sending it out, getting it answered, getting it back, changing it. But now we've got a good relationship with the wound nurses and they understand what we need. So, it's become a lot easier. C101 I think we have to work collaboratively together. We really do need to have a larger voice because it impacts on data, it impacts on funding, it impacts on decision making across everywhere. We want to ultimately improve patient care, that's the end goal. So, we need to really work together. Data is important. I think if there was one message, data is so important, so let's try and get it right. C103
		Following the coding standards and coding guidelines	We use the Australian coding standard; the standard reference number is 1221 and also Victoria have their own set of rules that go with that as well so using a combination of the two. From that there must be documentation by a clinician or a nurse with evidence of assessment of the pressure injury and commencement of a treatment plan. C201 I don't think I code it as often as you perhaps want to code it. Yeah, because the documentation is probably as such that it's only showcasing routine skin pressure area – care, rather than something then that the documentation meets the standards that I use to code. So that is a standard principal and the additional diagnosis standard. There're certain parameters that we need to make sure are documented before we can code a principal diagnosis and then so often if the pressure injury is more than not an additional diagnosis, there's quite a few checks that you've got to make sure are documented before that diagnosis actually meets coding criteria and that's where the difficulty lays. C403 There're the Australian coding standards; and then adding to that, the Victorian – Victoria decided to create additional diagnoses criteria, so that's like an add-on to the coding standards. And then we also have the [Vic] coding committee, which people can write in coding queries, and then they're answered, and they essentially become rules that you can – advice you can follow. And then there's the national body, which are the national coding rules. Which again is the same sort of principle, people can write in coding scenarios and they will give advice on how best to code them, and then they're considered advice that you need to follow as well. So, if there's a particular coding rule that says, “In this situation you need to code it like this,” you have to follow those rules, you can't just then change your mind and do something else. C401 So, the Australian Coding Standards is quite clear about the coding of pressure injuries. And together with the ACS 0002, which describes what is necessary for the standard of increased clinical care, which allows you to code it as an additional diagnosis, it's really very clear. There's no ambiguity about the coding of pressure injuries. Ambiguity comes in the clarity of the clinical documentation. C203 For example, sometimes a patient will come in with a pressure injury and it can change some stages. So, from a stage 1 to a 3 to a 2, but we always code to the highest stage. C101 I've got to be extra careful with the prefix, whether that was a pre-existing condition, or developed while the patient was in hospital, because that's one of the Australian Commission on Safety and Quality in Health Care hospital acquired complications. C303
		Professional development	I'm always interested in anything that will help me improve my coding and my knowledge. Whether that's a pressure injury or another condition. C101 Each time there's a coding update we all do it; and it's been online now, so we all do anything that's mandatory, for sure. C205 Twice a year there's a whole day coding workshop. And then we have a coding quiz every month. And then, you're supposed to have an hour a month to read all the new advices. C302 I have done a workshop a few years ago when the grades of the pressure injuries were first introduced into the coding system. C404
		Involvement in health professionals' education	Sometimes, it is about just educating people on the type of code that we use, the definition behind it, what the code is, if the definition has changed over time. So, we definitely do educate them on that. C103 I don't have a title. It's just part of being a clinical coder. Almost everyone has additional jobs in reporting or education. C203
Knowledge	Knowledge	Procedural knowledge	So, we get the electronic medical record and usually to look for a pressure injury. We look at the wound nurse notes and then we extract from their notes in the software that we use at the [health service] … In contacts, we look at the whole medical record or the admission notes. So, sometimes pressure injuries can be documented by nurses, medical staff, podiatry, but we usually code in to the wound nurses, because that's their specialty. So, we have to extract the location of the pressure injury, the level, so the stages. Also, whether it was acquired in care or hospital-acquired or whether it was present on admission. Then we go into software called 3M Coder and we code based on that information that we've got. C101
		Knowledge of the ICD-10-AM classification	The company that actually produced ICD 10, the Independent Hospital Pricing Authority, IHPA… they release coding advice and education on a quarterly basis… So, we make sure that we read through all of those when they come out, or at least all of the information that's relevant to the coding that we do. C404 Q: How would you rate your knowledge on the information contained within the ICD-10-AM, the International Classification of Diseases? A: I'd say it's pretty good. Again, I've had a fair bit of experience, so yeah. I still have to refer to the standards and review things every now and then, but yeah. C201
		Knowledge of pressure injury classification	But we do have some coders who have a nursing background and then done their studies and become a health information manager. So, my team leader was actually a nurse, so she's got a lot of background knowledge and she understands a lot of the concepts a little bit better than I do. C101 Australian Coding Standards, ACS… So, we use those definitions. And they also – not only have clinical information in how to classify pressure injuries, they have a section called pressure injuries. They also have a section about condition onset flags. So that's determining if it's present on admission or occurs after admission. So, we use those definitions as well. Our ACS is pretty good explaining the pressure injury staging. And we've had in-house education sessions. So, again, [I could rate] my knowledge of probably a nine [out of ten] for me and our educators maybe a seven or eight [out of ten] generally. C102 Q: And how would you rate your knowledge on pressure injury classification and staging and skin changes terminology? A: Well, our wound care chart has lovely pictures on it. So, really that's my education about it. The more ugly the wound – usually the higher the stage. But yeah, I certainly – I'm not off the top of my head, I wouldn't know what the definition of a stage 1 vs. a stage 2 vs. a stage 3 is. I don't have to know that to decide what code. I just need the stage documented and then I'll go with that. C202 When I became more involved [in coding] through the years, it's [the pressure injury classification] certainly changed a lot, the coding of it; and when it changed, I really went into reading the descriptions. And, where I've worked, at various places, the wound chart really goes into describing the level. So, I've read a lot, looked at photos as well, if I can, because that really helps to understand the severity of it [pressure injury]. C205
		Impact of COVID-19 on training	We were a bit restricted last year with group meetings with COVID, but we did more online, so she'd [the coding educator] send out quizzes on a regular basis that everybody had to complete. And she records their answers on a PowerPoint presentation on a topic. So, a bit of a mixture of approaches. C202 The manager has done education sessions with the consulting physicians here, but again that was before COVID, which it hasn't really happened in the last year, but that is something that they talk about, how critical the accurate documentation is and a documented plan of care. C203 So back pre-COVID days, we'd actually get, say, the skin integrity nurse to come and we would – they would talk to us about their process and we would talk to them about our process as well to try and bridge that gap between understanding the care delivery process and the documentation process and then the coding process, so - and linking those three areas together. C403
		Knowledge: suggestions for improvement	Q: Is there any improvements that could be made to ensure accuracy of coding? A: I think it comes down to documentation and just continuing to educate [clinicians] when we can. It's kind of hard if you don't know what to do or why it's needed, then you never do it. But if we can get it out there, like we have with the pressure injuries and with the wound nurses then we can see the improvements and get better documentation and better data and coding. C101 I like learning the anatomy about the different [pressure injury stages], like what makes a stage one a stage one and the definitions from a clinical perspective. But also, I would really love some ideas to take back to our CNCs on other organizations that have an electronic record and their data flow sheets, all their wound charts and etcetera, so we can see what else is out there and potentially improve what's in our system. C103 I think apart from the documentation the other thing and you were asking about before with our knowledge of pressure injury terminology and things like the skin changes and what the staging actually means and the progression of the injuries. C201 I think webinar's good, and it would be great to get a variety of different scenarios, or different people, different treating clinicians perhaps, and maybe different sites so that we can see how different sites do code and find the documentation, or any issues that they've come across or resolved. C401 Although you watch the presentation and you're actually on board and you listen, sometimes, you don't catch everything or you don't understand everything. So, what I've done is, on my own time, I've gone back in and just re-watched the presentation, just sit and taking a few of my own notes. C104 I like face-to-face workshops, but online webinars and things well we've seen a rise of that kind of thing in the last year due to pandemic. So that is a good way of being able to capture everyone at a time that's convenient for them to do it. Whereas face-to-face workshops are more difficult when you have part-time staff, etc. Yeah, so I guess webinars are a good way. C202
Behavioral Regulation	Action planning	Ensuring accuracy of coding	Q: How do you ensure the accuracy of the code? You touched on it a bit before… A: Yeah, the description in the code or the codes for say the area and the stage, I would not just click on the code, I would go into the tabular list which gives you more detail of the area because the most common ones are probably on the heel or the sacrum but, sometimes, you'll get the malleolus or something like that and I think oh gosh, I'd really better click on the tabular list to really look at the whole definition of this code, just to check I've got the right stage and body area. C205 Q: Now, we're just looking at the accuracy of coding. How do you ensure that the patient episode is allocated to the correct DRG with pressure injuries? A: Obviously, the first thing you have to do is you have to make sure that the principle diagnosis is correct, and then that will usually determine what DRG it falls into. And then if they have a procedure, the procedure may change the DRG, or if they were admitted for a particular procedure like a hip replacement, or an appendicectomy or something like that, that will determine what the DRG is. And then, in terms of the DRG split, so C, B, and then A DRG, that will depend on the complexity. So, for example, if they do have a pressure injury that's treated, that might impact the DRG. C401 A: So, we read podiatry in-patients notes, especially for pressure injuries of the foot or toes. Q: The wound chart? Would you double-check that? A: Wound chart, yes. Wound chart, nursing notes, podiatry and obviously the medical in-patient notes as well. C101 We do have a hybrid model here at [private healthcare], where we use the PAS, the Patient Administration System, in conjunction with a paper record. But all the nursing notes and the doctors' notes are handwritten, which obviously takes more time to decipher. There are often doctors letters, which come up on PAS or in the correspondence section, which aren't typed. We do need to do significant work on getting discharge summaries because the rate is very low, which also is an excellent source of information when it's there. And our wound charts… the way that they're set up is very difficult because we cannot code pressure injuries off the wound charts because they don't provide sufficient space for a written assessment and a plan of care because they're basically tick charts and body shapes with diagrams that they fill in to indicate the place of the injury or a device and don't meet the coding standards to allow us to code from those charts. And then often it's not backed up in the notes; and that's where we come into problems with the documentation. C202
		Documentation query	If there wasn't all the information that I wanted and there was a DRG impact to that particular admission, then I potentially would need to send a query. So, a documentation query is when we send a question to the clinician with all of the available documentation that is in the record and we ask them. For example, when I say clinician I might send a pressure injury query to our pressure injury CNC nurses. C103 But we've had a policy here in – well an instruction to the coders here in the past that we generally don't query things that won't make a difference to revenue. So, if you've already got that episode of care into maximum revenue then there may be no need to query the pressure injury. If that was the diagnoses that was going to make a difference to revenue then yes, you'd definitely be querying it. C202 [In relation to pressure injury] And you can see that I haven't got quite the full picture here, and I need to put a doctor query in, and the funding will be improved, then you would go ahead and put a doctor query in. If I've got a patient admission that I'm coding and I can see perhaps there is a pressure injury in there, but coding it doesn't increase the funding, I wouldn't then ask the doctor for more information or skin integrity specialist for more information because it's not going to cause any difference. And that's where the gap is because it's public health, and you would only do that to help optimize your funding, so you can make sure that you're reimbursed for that episode of care, but if you are not going to optimize the funding, you wouldn't put the query in, so – yeah, that is a bit of a gap. C403
	Self-Monitoring	Double-checking codes	When I open a record or an episode, I write down anything that would meet the criteria for coding; and then I signal out anything I need to check and I would put the codes in the 3M Codefinder [health information system] and then I'd go and do that whole process again just to double-check and then I check the DRG before finalizing it just to ensure that say it's not an ear, nose and throat DRG with a completely laparoscopic cholecystectomy. So just check that it's relevant, the DRG matches the case mix and the codes. C203 So, I might pull it up myself and go, “oh my God, that one wasn't an endoscopic one, it was a non-endoscopic one, so it's coded wrongly.” So, yes, I do little things like that for my own purposes, so that I can get the coding quality correct. So yeah, there are a few simple audits yes that I do pull, but the research and epidemiology tool, is somebody else's portfolio. C402 So, you do, definitely do a check before you hit enter. C403
		Using systems to ensure the quality of coding	So, we ran a program called PICQ, which is a Performance Indicator of Coding Quality, so that software – well actually we upload a file, an extract file, each day to [the name of the company], which is the private company that owns that software. So, all our coding every day gets run through that; and then each coder gets an email the next morning if they've generated an error that's been picked up that way. So that's probably more quality check. C202 We use a PICQ error program which picks up our errors and gives you a report every day if you have a warning or an error from the previous day and you can go and clarify the record immediately while it's still fresh in your mind. And besides with correcting the record, it also helps you learn about what triggered that warning in the first place and how you may avoid that in the future. C203 We also can run our own reports using Quick View software. So, on a monthly basis I'll run a report on that to see how many HACs [hospital-acquired complications] we had; and so, if it looks like an unreasonable number, we'll pull records out and check the coding. If it looks reasonable, do a quick desktop audit to make sure it makes sense. I guess that's the main way, monthly reports. C202
Intentions	Stability of intentions	Direct impact on the patient's episode of care	But I mean, the reason why we're coding is for a summation of that person's journey. A person can have a variety of issues which weren't treated; so, we shouldn't be coding them, and it hasn't impacted that person's stay. I think that the discharge summary, if done correctly, have the most important diagnosis available. C103 You have to look at each episode on merit, you can't really go back to previous – If you've got a patient who's been admitted 30, 40 times with the same things, you still have to take each episode on its own merit. C401
		Optimizing patient's funding	Q: And would a pressure injury capture and coding make a bit of revenue? A: Depends what the patient's in for originally. Sometimes, it will make no difference. And it just depends what DRG [Diagnosis. Related Group] the episode's in the care. I mean, sometimes, the diagnosis can make the difference of $10,000 between if something's coded or not, but it's not always a pressure injury to make a difference and it depends on the DRG. Sometimes, you can have the same code and it will affect one DRG, but it won't affect another DRG. Depends on the complexity level that's been assigned to it for that visit. C202 Q: So, you'll come across information that might say it looks like a pressure injury. Do you then have to send out a query about that? A: It depends on the funding that that patient is having. If it's going to make a difference to the funding, then we would send out a coding query about it. But if it's not going to affect the DRG, the diagnostic related group, then we don't spend time on sending a query. That's only appropriate for the funds that use DRG funding. Some funds are funded by diagnostic related groups, and the more detailed documentation, the higher the split. And of course, we want to reflect as accurately as possible everything that happened with that patient because it will change the amount of funding that the hospital receives. So, another patient that's on a per diem rate and we receive the same amount per day regardless of what's wrong with them, we would spend less time following up and chasing documentation to accurately reflect what is already written in the record. Q: Because you've already got them under a daily funding. C203
Skills	Practice	Supervised practice	You do a year training so where your records are being checked, your coding's being checked and you're learning the different specialties of the hospital. I'd probably say [it takes] maybe about 4 years to be really confident. C201 We do have an extensive training program, but we don't have new coders at the moment, because it does, it takes a fulltime person to train them and things. And I think every hospital in the state though, really, they get – if you're not experienced, it's very difficult at the moment to get a job, because none of the hospitals will put on a trainee. But just because you finished Uni, does not mean that you are set forth on coding – it's another 2 years of being on the frontline. C402 And, I think, that's why the training goes for a year because – I might be coding a respiratory case, but they've also got cardiology and renal as well. So, you've got to really be trained in every area before you can [code on your own] – “Okay, you're off training now, out you go, you go code on your own,” and not every case of mine was then checked then because I had then proved a certain level of ability in that year, yeah, as you cover everything. And as you get signed off on one, then if you do, do some live coding, it's only for those renal episodes. So, it's – they're quite careful that they don't release you until they're confident that you've been upskilled in all the areas. So, it's nothing like having theory. Theory's great, but in actual practice, it's knowing the inhouse systems, it's a completely different kettle of fish. C403 And once you – you do lots of practice coding, so you're coding ones – cases that have been previously coded, so like shadow coding them. Then you start coding some live ones and all of your ones that you've – say, I've just freshly learnt about renal, then I start coding my own renal and they're all checked themselves and then you have to pass – the trainer has to review and you have to pass that unit before you can move on, so you have to be able to prove some ability in making sure that your code is matching their coding and they're quite confident that you can go ahead and keep coding that unit. C403
Beliefs about Capabilities	Perceived competence	Perceived competence	Q: How would you rate your knowledge and skills related to pressure injury coding? A: On a scale of one to 10, 10 being brilliant…Coding, I would hope to be a 10 out of 10. C103 I've been coding for over 20 years. I am a coding advisor at where I work, so I actually am a point of call for other coders to ask questions of. I have coded consistently across that 20 years. I have worked at a number of different places. So, fairly familiar with all different types of documentation, always stay on top of the education, and always reading new queries that come out. C301
	Self-confidence	Self-confidence	Q: How confident do you feel allocating pressure injury codes to patients' period of care? A: Depends on the admission. Again, I guess going back to just the scenario of that patient's stay and the way it's documented so sometimes it's quite easy, and I feel very confident, other times it might be a bit harder to try and work out whether it's appropriate to assign it. In that case, I'd then discuss it with another coder and then from that discussion feel pretty confident once I've had a second opinion. C201 If I've got that sufficient documentation, then I'm confident to code it. There are times where sometimes I have to think really, really hard whether or not it meets additional diagnosis in that particular case. So actually, maybe I would say 70% of the time I would be comfortable with assigning that code. C103 I'm pretty confident when there's information or when there's even the word ‘wound’, I feel like I'm confident in finding out more and what they mean and a ‘wound’ doesn't necessarily mean acute trauma. I like to look deeper to see what is that wound, so I feel quite confident in my own practice. C403 We're very confident. We all have our senior staff who we report to, as in senior educators. If I find something that is inaccurate or I'm querying it or I'm not sure about, I'll just ask her. And I will just send her an email. And we always give feedback. And therefore, our educators tend to have meetings and discuss anything that comes up, or anything that's new, or anything that we find that it's unusual. C104
Memory, Attention and Decision Making	Memory	Memory	Sometimes it's worse the longer you've been coding because you remember five/ten years ago how we used to code it and that kind of got stuck in your brain, but the more recent stuff didn't. The newer coders might refer to the standards are bit more often because they're used to doing that in their training and they're kind of aware, whereas one that's been coding in years or so might think oh yeah, I know that and not go back to it as frequently, so may still be depending on memory. C202 People would go shortcuts because they start remembering codes off the top of their head, people will always find shortcuts, but it's that self-checking that you need to make sure that, “Okay, I've coded this.” C403.

### Environmental Context and Resources

All 16 coders discussed the accuracy of hospital record documents as the main factor that ensures the accuracy of PI coding. The following information needs to be included in the nurses' notes: (1) definitive PI diagnosis; (2) PI stage; (3) PI location; (4) if PI detected on admission or acquired in hospital; (5) PI assessment conducted; and (6) the PI care plan. If this information was included in the discharge summary, coders were expected to confirm this from nurses' notes. However, sometimes nurses used incorrect terminology or incomplete description; for example, the patient record may indicate “injury,” but it would be difficult to determine if it was a PI, thermal injury, or injury to skin sustained as a result of radiation therapy. Sometimes, clinicians used terminology such as “skin lesion,” “blister,” and “wound” instead of “pressure injury,” which required further clarification. Nursing notes with correct terminology was particularly important for complex cases associated with chronic wounds, including venous leg ulcers, diabetic foot ulcers, and PIs. The main suggestion for improvement of the coding process provided by all participants was “to improve the accuracy of documentation.”

At the time of the interviews, three public health care services had transitioned to the electronic medical record system (EMR), while the private health service was using paper-based records. The coders identified that the transition to EMR might have impacted quality of coding. For example, if nurses used an incorrect terminology for PI, this may not even appear to coders in the EMR. Also, from the coders' perspective, the EMR made it difficult for people documenting a PI to find how other clinicians had documented the PI during the patient's stay. One coder (C302) reported clinicians “had more freedom” using the paper-based form and were able to include drawings; whereas, electronic entry of PI information was more structured and did not allow for this “freedom.”

### Social/Professional Role and Identity

Coders reported internal and external audits are the main facilitators of high-quality coding. Participants acknowledged that internal audits are conducted by coding auditors primarily for revenue purposes, but also help to improve the quality of coding. There are also reconciliation audits; and some health services have PI-specific reconciliation audits to double check that “the prefixes are correct,” that is PI detected at admission and HAPI were accurately reported. These audits are usually followed by an education session for the coding team.

External audits are conducted by the Department of Human Services and by private health funds. The private health funds conduct two types of auditing. Pre-verification auditing is conducted to check the codes and related evidence prior to a health fund paying the hospital for that admission. The other type of auditing is conducted yearly and includes external auditors coming onsite and selecting about 200 records for review, as participant C202 explained.

All participating health services have coding educators/advisors, available up to 5 days a week, to provide support and training for coders. A request can be made via the Coding Advisor software; and the required guidance and standard recommendations will be provided upon request. Further, coding educators run intensive training programs for interns and new staff. Coding educators also inform the coding team about all recent changes to standards and monitor if these changes have been implemented.

In pre-COVID time, internal meetings were used to answer frequently asked questions and to discuss changes in coding standards. The internal meetings also provided an opportunity for coders to communicate shortcomings in medical notes. However, at the time of the COVID-19 outbreak, most coders moved to working from home and did not have the opportunity for face-to-face internal meetings.

Interprofessional collaboration also impacted data quality and subsequently the quality of PI coding. Regular meetings of coders with wound nurses improved nurses' understanding of what needed to be documented. Coders explained to wound nurses that it was easier to work when they had well-documented cases, which reduced the number of queries and saved time. Most coders reported that the quality of coding impacted on data, funding and decision making, and that this ultimately improves quality of PI data and therefore improves patient care. For example, coder C203 said: “We have done some education with the nursing staff in order to help empower them to really know how the documentation is going to affect the patient's coding and ultimately care in the hospital, how much the hospital can provide care, and that has resulted in some improvement here in [healthcare service].” The participants acknowledged that when clinicians say that they are too busy to meet with them, coders always try to convince clinicians that better explanation of what should be documented will save clinicians a lot of time, they would spend answering coders' queries.

Following the coding standards and professional guidelines, ongoing professional development, and involvement in health professionals' education were the main professional roles discussed by all coders. They acknowledged following national and state coding standards and reported that coding standards were clear on how to code PI; for example, coding standards specified clearly how and when to code principal and additional PI diagnosis. In addition to these standards, there were coding scenarios developed by the coding committee, which also provided advice on how to code scenarios. For example, coders reported they always coded the highest stage of PI and all parts of the body if more than one PI were identified. Coders reported they paid specific attention distinguishing a PI on admission and HAPI.

Moreover, participants acknowledged the need to be up-to-date on the changes in coding standards in order to successfully implement standards into practice. They also acknowledged the need for researchers to interpret statistical information in light of the coding requirements for a particular year and to compare data across different periods of time with caution. They provided an example on how changes in coding standards affected the availability of statistical information on HAPI over time, which could easily be misinterpreted as improved quality of care:

“They change the goalposts regularly, and it's very hard for us to keep on our toes. As far as you're concerned with pressure injuries is that you may have found a period of time from when ACS 0002 [Australian Coding Standards 0002], which was not this edition, it was the previous edition…There would have been a period of time where pressure injuries weren't– appeared to be coded less because we were looking to documentation of a care plan being carried out, not just the documentation of a care plan. So, there probably would have been a drop in pressure injury coding, a significant drop from when ACS 0002 came in, you will have noticed. So, if you looked at data from maybe 8 years ago, you would have seen pressure injuries going nuts, like people just coding them left, right and center. I've got to look at the years on my books.” C301

The need for ongoing professional development was another professional activity identified by all coders who reported it was mandatory on a yearly basis. Regular face-to-face workshops were attended pre-COVID. As part of professional development, the coders reported completing online quizzes on a monthly basis. They also had specific online training on the ICD-10-AM classification every 2 years, to review new editions of the PI classification. In addition to mandatory training, coders attended workshops related to specific health conditions, including PI. Coders acknowledged that the COVID-19 outbreak impacted educational sessions. Some coders reported regular face-to-face group meetings were replaced with online meetings during the pandemic. In some healthcare services, online quizzes were developed for coders to be completed online; and answers were summarized and distributed to all coders via PowerPoint presentations, with correct answers and explanations. Some coders reported that their collaborative sessions with consulting physicians and skin integrity nurses ceased during COVID-19 outbreak because clinical work was prioritized during the pandemic period.

Six coders, who indicated their current position as coding educators/auditors, and some regular coders discussed their involvement in coders' and clinicians' education as their professional activity. One of the coders, C203, explained: “I don't have a title. It's just part of being a clinical coder. Almost everyone has additional jobs in reporting or education.”

### Knowledge

The participants discussed their procedural knowledge of the coding process, knowledge of the ICD-10-AM classification, and knowledge of PI classification. They also provided suggestions on how to improve their knowledge, including the topics of interest and preferred learning methods. When probed regarding their need to develop knowledge further, most coders suggested that coders from a non-clinical background and less experienced coders benefited from webinars on PI classification. Other suggestions were to improve coders' knowledge on how and where PI was documented in the electronic medical record and to have various clinical case scenarios with reflection on coding – ‘it would be great to get a variety of different scenarios, or different people, different treating clinicians perhaps, and maybe different sites; so, that we can see how different sites do code and find the documentation, or any issues that they've come across or resolved’ (C401).

Considering that most coders interviewed had extensive coding experience, they had excellent knowledge of PI classification. They discussed the following steps of the coding process, including (1) accessing the EMR/or paper-based record and reading the wound nurse's/other clinicians' notes to confirm the diagnosis of PI; (2) accessing the whole medical record or the admission notes; (3) extracting the location and the stage of the PI; (4) referring to the Australian Coding Standards to confirm that the PI documentation obtained meets criteria for coding; (5) determining whether a PI was present on admission or acquired in care; and (6) coding PI based on the acquired information using 3M Coder software. Most coders described their knowledge of the ICD-10-AM classification as “good” and “above average” because they are proficient and regularly follow the two-yearly classification updates.

Clinical coders from a nursing background with clinical care experience reported they have excellent knowledge of PI classification. Clinical coders from the private health service, who had worked with paper-based records, reported they took information from the wound care chart, which included photographs clearly depicting different stages of PI. Other coders reported they used the PI classification provided in the Australian Coding Standards, which they found to be clear and informative. A few coders rated their knowledge of PI stages as “not so good,” particularly when differentiating unstageable and suspected deep tissue injury. Also, they said that they “don't have to know” the classification of PI to decide “what to code,” and just need to code “the stage documented” in the notes because “coders are not allowed to diagnose” and use stage of PI as documented by clinicians.

Various suggestions regarding the modes of delivery were shared by participants. Some coders preferred face-to-face workshops because of the ability to ask questions, but also acknowledged that this mode of delivery would be impractical during COVID-19 and post-COVID-19 periods. Many coders agreed that online modules and webinars would be their preferred mode of delivery because the online module and the webinar recording could be accessed at any time. The participants also preferred clear, concise and straight to the point content, and coding training sessions no longer than 30–60 min.

I like face-to-face workshops, but online webinars well we've seen a rise of that kind of thing in the last year due to pandemic. So that is a good way of being able to capture everyone at a time that's convenient for them to do it. Whereas face-to-face workshops are more difficult when you have part-time staff, etc. Yeah, so I guess webinars are a good way. C202

Coders reported the benefit of inter-professional collaborative workshops, where nurses and coders shared their perspectives. Collaborative workshops had the potential to improve coders' knowledge of PI classification and clinicians' understanding of what information should be included when documenting PI in the medical notes. Specific emphasis was placed on the care plan – ‘there has to be some sort of a care plan for it [PI], for us to code it. There has to be a specific care plan, to see that it's been assessed. And a care plan's been put in place, and that care plan has been implemented… but a bit of cream on a red bottom doesn't cut it anymore’ (C402).

### Behavioral Regulation

To ensure the accuracy of coding of PI stage and the body area in which the PI occurred, some participants said that they would always “go into the tabular list which gives you more detail of the area” rather just simply “clicking on the code.” Accessing complete set of details allowed them to ensure that the right PI stage and the right body area was selected. To ensure the accuracy the Diagnosis-Related Groups (DRG) assigned during coding, coders ensured that the principal diagnosis was correct, which determined the DRG. So, for example, if the patients “do have a pressure injury that's treated, that might impact the DRG” (C401).

Coders used multiple sources of information to ensure that the reported PI met the coding standard to improve coding accuracy. This included checking the wound chart, discharge summary, nurses' notes, podiatrist notes, and the medical in-patient notes. As one of the coders (C202) reported: “we cannot code pressure injuries off the wound charts because they don't provide sufficient space for a written assessment and a plan… and don't meet the coding standards to allow us to code from those charts.” They also pointed out that if the wound chart information is not backed up in the nurses notes and discharge summaries, coders would need to initiate a documentation query.

If there was incomplete or inaccurate documentation, coders initiated a documentation query to a clinician. They would usually send coding queries with all available documentation either to the unit doctor/specialist or to a clinical nurse specialist. This process usually took the coder about 15 min, although it could take longer depending on the case complexity. Clarification then would take a couple of days depending on clinician availability. All of the participants affirmed that they only initiated documentation query when it impacted whether funding would be improved. They reported that querying the clinical doctor regarding documentation was mainly used for optimizing funding. Often coding a PI would not optimize funding and, therefore, they would not initiate a clinician query “because it's not worth the time and effort of the clinician, it's not going to bring any more money back to the hospital, but you're then making a gap with the quality side of things” (C403).

The participants discussed ensuring quality of the coding they did through simple “self-audits” that double-checked the codes before data were entered. As one of the coders (C203) discussed, she repeated the whole process of coding at least once to double check the codes that she used in the 3M Codefinder health information software, and she checked the DRG matched the case mix and codes in patient information. Another coder (C402) said that, from time-to-time, she realized that incorrect codes were allocated, and she would “pull up” the case and check if the codes were correct.

In addition to simple checks, coders also used the computerized systems to ensure quality of coding. They said that an extract file of their coding for the day would run through the Performance Indicator of Coding Quality (PICQ) error-picking software. If an error was picked up by the system, the coders would receive an error notification message. The record would need to be clarified, and the coder would then deal with.

Coders also said that they can run their own reports using Quick View software on a monthly basis. They would usually run a report on the number of hospital-acquired complications they had over that month. If they found an unreasonable number of hospital-acquired complications, they would pull records out and check accuracy of coding: “If it looks reasonable, do a quick desktop audit to make sure it makes sense. I guess that's the main way, monthly reports” (C202).

### Intentions

Coders further explained that they coded only health issues that had a direct impact on patients' episode of care. Patients usually presented with a variety of health issues, not all of which would be the focus of treatment. Health issues that had no direct impact on the patient's episode of care were not coded. If the coders had incomplete information on PI upon admission and the PI did not progress during hospital stay, coders would not initiate the coding query process because the PI should not be coded.

The reason why we're coding is for a summation of that person's journey. A person can have a variety of issues which weren't treated; so, we shouldn't be coding them, and it hasn't impacted that person's stay. I think that the discharge summary, if done correctly, have the most important diagnosis available. C103

The participants said that their intention to code PI and initiate a coding query was also dependent on the financial outcome—the ability to optimize patient's funding. They reported that, sometimes, “the diagnosis can make the difference of $10,000, if something's coded or not” (C202), depending on the level of complexity assigned for a particular admission. They said that they “want to reflect as accurately as possible everything that happened with that patient because it will change the amount of funding that the hospital receives” (C202). However, if it is not going to optimize patient's funding, they would not “spend time on sending query” (C203). As the coders further explained, this is only appropriate when funds require use of the Diagnosis Related Group (DRG).

### Skills

Years in practice and importance of the supervised practice were discussed as main factors that influence the quality of coding. Some participants openly said that university training alone is insufficient to start independent coding, and it takes from 2–4 years for a graduate to develop the necessary coding skills. They discussed the importance of in-service training program for graduate coders and supervised practice for developing their skills and learning the coding process of the different hospital specialties, including PI.

So, at uni [university] there was a unit based on medical terminology. But you learn on the job as you go. C101

Okay, someone completely green from uni [university], I would say would potentially take about 18 months to train. I have to reiterate also that seems like a long time but we have a very complex case mix at the organization that I work with. C103

You do a year training, where your records are being checked, your coding's being checked and you're learning the different specialties of the hospital. I'd probably say [it takes] maybe about 4 years to be really confident. C201

Some participants used the terminology of “live” and “shadow” coding. Shadow coding was described as coding cases that were already coded as part of the supervised practice program, while live coding was described as independent coding of live cases. They explained how each coder was required to complete and pass a particular specialty unit before being allowed to independently code that specialty. Ongoing skills development has already been discussed as part of “professional role” in the Social/professional Role and Identity Domain.

### Beliefs About Capabilities

Most coders we interviewed perceived themselves as competent. Some were experienced coders who had worked as medical coders for decades at various health services. They were confident in allocating PI codes, sending queries, and supporting junior coders.

I've got a lot of coding experience and as soon as I see the word it's like a beacon goes off in my brain and I make sure I find out as much information as I can about pressure injuries because they can be catastrophic to patients. It's important to capture the data. C205

I've been coding for over 20 years. I am a coding advisor at where I work, so I actually am a point of call for other coders to ask questions of. I have coded consistently across that 20 years. I have worked at a number of different places. So, fairly familiar with all different types of documentation, always stay on top of the education, and always reading new queries that come out. C301

They linked their confidence to knowledge of the process of coding and ICD-10-AM classification. Some coders came from nursing backgrounds and were confident in their own evaluation of PI staging. Junior coders explained their own confidence in themselves as coming from the support available to them from coding advisers. Most coders acknowledged that if they had sufficient documentation, they were confident to allocate a PI code.

I've worked in aged care for such a long time. I feel like I've got that bridge understanding between delivering care and understanding what we need to document to make sure that we can keep delivering that quality care because if you're not documenting what you've undertaken and done, there's no way you can assess and re-assess and then create practices moving forward to make sure optimal care and person-centered care are delivered. C403

### Memory, Attention and Decision Making

Coders discussed that, with time, they memorized the codes and used them “off the top of their head,” as C403 explained. In this case, they said it is important to double-check that back in the index to ensure the quality of coding: “But there is a standard on pressure injuries. So, if I needed to refresh my memory, I could certainly read the standard in the book, if I was a bit studious about something” (C402).

Some coders acknowledged that it could be difficult to reconstruct their procedural memory when new changes were implemented. This is particularly problematic for coders who were in service for a prolonged period of time, while recently graduated coders would frequently consult the Standard and the guidelines. In general, if they needed to refresh their memory of codes, the ICD-10-AM classification, and the PI stage, coders said that they would either use a search engine or book a workshop.

## Discussion

National Safety and Quality Health Service Standards use coded clinical data for monitoring patient safety through its hospital-based outcome indicators (25); HAPI has been identified as an important indicator of the quality of care (35).

One of the main findings was that hospital coders often lacked vital information in clinicians' records needed to code PIs and report quality indicators accurately. Coders identified frequent need for additional information on (1) whether a PI had been diagnosed; (2) the PI stage; (3) PI location; (4) if the PI had been detected on admission or acquired in the hospital; (5) how the PI assessment was conducted; and (6) the subsequent PI care plan. If this information was included in the discharge summary, it was also expected to be confirmed in the body of the nurses' notes. The described efforts to “improve the accuracy of clinical documentation” are consistent with other studies conducted in Australia (25) and specifically on PI (1, 2). Studies conducted in Canada (29, 30, 36), Portugal (37), UK (38), and USA (39) also highlighted the need for quality improvement processes for PI clinical documentation. Nursing documentation improvement is a vital component of the complex capacity building programs on PI prevention in acute care services and is relied on by coders (40, 41). Educational interventions on the quality improvement designed for nurses have the potential to improve the quality of PI documentation (42). Studies in other medical fields suggest that both paper-based (43) and electronic records (44, 45) need to be improved. However, PI reporting was found to be more accurate and complete in the electronic health records compared with paper-based records (46).

Our findings identified three methods of quality assurance were important to coders to ensure accuracy of PI reporting: (1) training prior to initiation of coding activity and (2) continued education, and (3) audit and feedback communication about how to handle specific complex cases and complex documentation. From a behavioral perspective, most of the coders reported confidence in their own abilities and were open to changes in coding standards. In general, coders expressed their greatest frustrations in identifying, from documentation, the appropriate information needed to apply coding standards. To assure the accuracy of clinical documentation, the proposed interprofessional collaborative educational sessions, which are in place in some health services were reported to be beneficial. Previous studies also reported that improved clinician-coder collaboration is beneficial (30) as it can improve the quality of coding (47).

Transitioning from paper-based to electronic records highlighted the need to improve training of both clinicians and coders. EMR implementation is a complex process, and clinicians have a critical role in successful EMR implementation (48). Documentation-related benefits of EMR implementation include timeliness, better quality and quantity of nursing documentation and improved quality of the documentation process (48). However, it may increase documentation time (49), particularly during transitional periods, when nurses have insufficient skills and knowledge of where to enter their notes on PI assessment. Moreover, services transitioning from paper-based to electronic records should ensure coders have full access and know where to access clinicians' notes on PI, and that they have appropriate training to access PI documentation on the EMR system.

Internal and external audits were identified as main enablers to ensure optimal coding, which is important for both the revenue generation and benchmarking of quality of care. This finding aligns with studies conducted internationally (50–52). NHS UK (51) developed a 10-points checklist to improve the quality of clinical-coded data, which includes (1) manageable levels of medical documentation and improved quality of medical documentation and easy to use EMR; (2) consistent and complete discharge summaries; (3) availability of the coding updating process; (4) regular engagement with clinicians; (5) regular analysis and routine audits; (6) attention to staffing issues, including the skill mix and the number of coders; (7) training and guidance; (8) the IT system used for coding are fit for purpose; (9) assessment units should be formalized to ensure all patient information is captured completely and accurately; and (10) broader uses, when clinical coded information underpins all aspects of health care management. In our study, coders identified the following personal approaches and institutional support systems to assure the quality of coding: (1) accessing the EMR/or paper-based record and reading the wound nurse's/other clinicians' notes to confirm the diagnosis of PI; (2) accessing the whole medical record or the admission notes; (3) extracting the location and the stage of the PI; (4) determining whether a PI was present on admission or acquired in care; and (5) coding PI based on the acquired information using 3M Coder software.

A few coders rated their knowledge of PI stages as “not so good,” particularly when differentiating unstageable and suspected deep tissue injury (DTI) because these two stages are more challenging to assess. The lack of PI stage differentiation skills is a common pitfall in PI staging and reporting that is discussed in literature (2). This finding aligns with the previous research on PI data conducted in Australia (1). Australian Coding Standards update to focus on unstageable and DTI PI two stages helpful as the coders reported using the standards to guide their classification, and they found the standards to be both clear and informative.

### Implications for Practice and Research

The Theoretical Domains Framework offers a comprehensive approach to studying the factors that influence routine use of professional practice. For hospital coders, whose work includes identifying and reporting PI data collected from hospital sources, the TDF provides a method for evaluating the barriers and enablers to ensuring quality PI reporting on a daily basis. Our results from interviewing coders in Melbourne hospitals identified both educational and feedback approaches that would lead to better quality reporting of PI. In particular, professional education in an interdisciplinary setting could help coders understand better how to apply clinicians' notes to inform the coding process. While, from a feedback perspective, improvement and tailoring of internal and external auditing processes would continue to improve PI quality. Coders were relatively confident in their own ability to apply PI standards, particularly if they had complete and accurate information in clinicians' notes, but expressed concerns about how to most effectively and efficiently communicate with the hospital staff on the importance of quality PI reporting. An in-depth exploration of clinicians' perspectives on documenting PI would offer a valuable insight into a collaborative practice that improves the documentation quality and consequently the quality of coded data. Coded data extracted from documentation in the patient's medical record is a vital source of PI data; and further research is needed to identify quality improvement strategies across countries and to facilitate an international consensus on PI data collection and reporting (53).

### Study Limitations

While we attempted to recruit clinical coders with different experience levels, most of our participants were experienced coders, who had worked in the field for 6 years and over and were in senior positions. Less experienced coders might, therefore, have a different experience of the process of PI coding. Our interview guide was loosely based on the TDF, and some domains and constructs might not have emerged for this reason. That is, we actively asked the participants to prioritize the domains of interest and did not prompt participants for less important domains, for example Emotions. We did not pilot the interview guide, although we did seek coders' input during the interview guide development. The transcripts were not sent back to the coders to verify the content. Although the opportunity to read the transcripts were offered to all participants, only one requested their interview transcript. In regards to transferability, our findings would be of interest to all countries that have adopted the ICD-11 and may be of interest to developing countries that have adopted a simplified version (54) of disease classification.

## Data Availability Statement

The original contributions presented in the study are included in the article/Supplementary Material, further inquiries can be directed to the corresponding author.

## Ethics Statement

The studies involving human participants were reviewed and approved by The Alfred Hospital Ethics Committee (Project No: 66/17) and the participating health services ethics committees. The participants provided their written informed consent to participate in this study.

## Author Contributions

CW, VT, and JB-H designed this research project and secured the grant. VT and CW designed the questionnaire. LT conducted the interviews. VT, LT, and CW developed coding framework. VT coded the transcripts, analyzed data and drafted the manuscript with support, and guidance from CW and JB-H. All authors critically reviewed the manuscript and approved the final version.

## Funding

This project was supported by the Australian Government's Medical Research Future Fund (MRFF) as part of the Rapid Applied Research Translation program through Monash Partners.

## Conflict of Interest

The authors declare that the research was conducted in the absence of any commercial or financial relationships that could be construed as a potential conflict of interest.

## Publisher's Note

All claims expressed in this article are solely those of the authors and do not necessarily represent those of their affiliated organizations, or those of the publisher, the editors and the reviewers. Any product that may be evaluated in this article, or claim that may be made by its manufacturer, is not guaranteed or endorsed by the publisher.
