# Supplementary material for: Clinical Coders' Perspectives on Pressure Injury Coding in Acute Care Services in Victoria, Australia
Source: Front Public Health. 2022 Jun 1;10:893482. doi: 10.3389/fpubh.2022.893482 (PMC9198603; doi:10.3389/fpubh.2022.893482)
Supplement: Supplementary file 1 [file Data_Sheet_1.pdf]

SUPPLEMENTARY FILE 1. INTERVIEW GUIDE

For Clinical Coders on Pressure Injury Surveillance in Acute Care Settings

PROJECT TITLE: Building capacity to optimise Pressure Injury prevention and surveillance across Monash Partners Healthcare services

SHORT TITLE: Building capacity to optimise Pressure Injury prevention and surveillance

1. **Introductions**
   i. Explain research (read plain language information statement)
   ii. Consent form
   iii. Gain permission for tape recording
   iv. Remind them – do not have to answer any question that makes them feel uncomfortable and interview may be stopped at any time; all names will be removed from transcript
   v. Any questions?
2. **Demographic Information - Could you start by telling me a bit about yourself…?**
   i. Year of birth
   ii. Gender
   i. Education (Bachelor in Health Informatics, Bachelor of Science - Health Science
   Other [specify]____
   Postgraduate courses [specify] ________________________________
   iii. Country of education (Australia or overseas-trained)
   iv. Occupation
   v. Duration of practice in hospital health informatics
   vi. Health Service
   vii. Period of work in this health service
   viii. Training courses on PI coding
   ____
3. **Potential Questions**

PI coding process

First of all, I would like you to share your experience of **PI coding**. How do you code PI?

Please allow your participants to share their experience. Do not interrupt. Later, you may ask the following questions:

- Can you please describe PI coding process?
- How long does it take to code PI?
- What software do you use for coding?
- Which reporting systems do you use to get information on PI?
- What definition do you use to code hospital acquired pressure injury (HAPI)?
- What policies and protocols on PI coding are available in your health care setting? What changes (if any) to coding have you had since the COVID-19 outbreak?

Accuracy of coding

- How do you ensure the patient episode is allocated to the correct Diagnosis Related Group (DRG)?
- What are the main pitfalls in coding PIs?
- How do you insure the accuracy of PI codes?
- How do you clarify inconsistent or non-specific information in a medical record, in regards to PI staging?
- Who do you usually contact if you need to clarify in regards to PI staging?
- What are the main shortcomings in the medical record in relation to PI?
- How do you communicate medical record content shortcomings?
- How confident do you feel to communicate medical record content shortcomings to management?

Knowledge and Skills

- How would you rate your knowledge and skills related to PI coding?
- How confident do you feel allocating PI codes to patients' current period of care?
- How would you rate your knowledge of PI classification and staging?
- How would you rate your knowledge of the information contained within the International Classification of Diseases, 10th Revision Australian Modification, commonly referred to as ICD-10-AM?
- How do you maintain ICD-10-AM coding books to make sure they are updated?

Education and support

- What support is available for coders in your workplace?
- What training courses have you attended on medical record coding and when?
- What PI training courses have you attended and when?

Teamwork

- Do you assist with the education of clinical staff with regard to ICD-10-AM coding of PI?
  Yes/no? If yes,
- How do you assist with the education of clinical staff with regard to ICD-10-AM coding of PI?
- Do you assist with medical research and casemix (patient statistics) projects?
  Yes/no? If yes,
- How do you assist with medical research and casemix (patient statistics) projects?

Current needs and suggestions for improvements

- Basing on your experience, what are the current needs of clinical coders in relation to PI coding?
- How do you ensure there is continuous improvement in ICD-10-AM coding and collection of quality health data?
- What improvements could be made to ensure accuracy of coding?
- What particular strategies could be developed to ensure optimal PI coding practices?
  Prompts: on individual level, on organizational level, on health system level, on policy level

Suggestions for educational courses

- What is your preference of educational activities on PI coding?

  Prompts: online, video link, webinar sessions, face-to-face workshop, time frame, refreshers/updates.
- In relation to online module, what potential topics could be included?

  Prompts: PI staging, PI reporting issues, PI coding issues?
- What would be your preferred completion time for online module (30 min, 1 hr)/ face-to-face workshop?
- What additional information you would like to include in this module/face-to-face workshop?

Thank you

Is there anything else you think would be important for me to know, but I did not ask you?

!!! Please ask the participant to connect you with clinical coders who might be interested to participate in this project.
